# Outcomes of retinal pigment epithelial detachment in Vogt-Koyanagi-Harada disease: a longitudinal analysis

**DOI:** 10.1186/s12886-022-02675-6

**Published:** 2022-11-19

**Authors:** Chuanzhen Zheng, Kaixuan Wang, Mi Zhang, Qingqin Tao, Xiaorong Li, Xiaomin Zhang

**Affiliations:** Tianjin Key Laboratory of Retinal Functions and Diseases, Tianjin Branch of National Clinical Research Center for Ocular Disease, Eye Institute and School of Optometry, Tianjin Medical University Eye Hospital, 251 Fu Kang Road, Tianjin, 300384 China

**Keywords:** Retinal pigment epithelial detachment, Vogt-Koyanagi-Harada disease, Central serous chorioretinopathy, Corticosteroid, Subthreshold micropulse laser photocoagulation, Pigment epithelial detachment angle

## Abstract

**Background:**

The aim of this study was to report the clinical profile and outcomes of retinal pigment epithelial detachment (PED) in Vogt-Koyanagi-Harada (VKH) disease, and to evaluate the correlation between PED and the subsequent development of central serous chorioretinopathy (CSC) throughout the whole corticosteroid treatment course.

**Methods:**

The retrospective study enrolled a total of 470 eyes with VKH, and 12 eyes with VKH and PED were recruited. Patients were divided into two groups according to the CSC onset or not throughout the whole course (the CSC group and non-CSC group). Best-corrected visual acuity (BCVA) improvement, and PED angle (PEDA, the angle between the two lines of the vertex of the lifted retinal pigment epithelium to the two edge points of the Bruch membrane) were compared between the two groups.

**Results:**

CSC developed at the site of the PED in 5 of the 12 eyes with PED, while in the remaining 7 eyes PED gradually resolved following therapy. The prevalence of PED and CSC in VKH was 2.55% (12/470) and 1.06% (5/470), respectively. BCVA improvement in the non-CSC group was greater than that in the CSC group, but without a statistical difference (*P* = 0.25). PEDA was significantly smaller in the CSC group than in the non-CSC group (*P* = 0.03).

**Conclusion:**

PEDA is an ideal parameter to reflect hydrostatic pressure and stretches for RPE. As PED predisposes to the development of CSC in selected VKH eyes, PEDA may be a valuable predictive factor for the development of classic CSC in VKH cases.

## Background

Vogt-Koyanagi-Harada (VKH) disease is an immune-mediated multisystem disorder characterized by bilateral panuveitis, neurological (meningeal), auditory, and dermatological manifestations [1]. The hallmarks of posterior ocular manifestations at the acute stage are bilateral multiple exudative retinal detachments (ERD), optic disc swelling, and choroid thickening [1]. Multimodal imaging examinations, such as fundus fluorescein angiography (FFA), indocyanine green angiography (ICGA), and optical coherence tomography (OCT), aid in diagnosis and change evaluations [2]. The mainstream therapeutic regimen is a rapid and aggressive high-dose systemic corticosteroid to suppress acute intraocular inflammation, followed by gradual tapering. Timely and aggressive systemic corticosteroids are the cornerstone of preliminary therapy. The subsequent oral corticosteroid should be gradually tapered off and sustained for at least 6 months to avoid a high recurrence rate and poor prognosis, at the same time, the initiation of immunosuppressive agents (such as azathioprine [AZA], cyclosporin A [CsA], mycophenolate mofetil [MMF]) has proven to be a great complement for corticosteroids in controlling the inflammation [1–4]. Moreover, some newer biologic agents (such as rituximab, infliximab, interferon alpha 2A, and adalimumab [ADA]) have been effective in small sample size of cases with poor response to both corticosteroid and immunosuppressive agents [1–3].

The anatomical separation between the basement membrane of the retinal pigment epithelium (RPE) and the inner collagenous layer of the Bruch membrane is known as pigment epithelial detachment (PED) [5]. Isolated serous PED is frequently associated with choroidal hyperpermeability on ICGA and is regarded as a transitional stage between pachychoroid and classic central serous chorioretinopathy (CSC) [6]. Nonetheless, serous PED is an unusual finding in acute VKH characterized by choroidal thickening [7]. Although VKH and CSC diseases are different in nature, they occasionally require differentiation because they share some common clinical features, including bullous serous retinal detachment on OCT and multifocal leakage on FFA. Sometimes CSCs can also develop in VKH patients [8]. Early diagnosis of CSC in VKH patients is important because the therapeutic strategies for these two diseases are contradictory, and adjustment of treatment is imperative once CSC occurs.

Few studies have investigated the clinical value of PED in VKH and its relationship with subsequent CSC development. Therefore, we conducted the first longitudinal analysis to describe the outcomes of PED in VKH cases and searched for a simple predictive factor for different clinical prognoses based on the PED contour. Hence, we hypothesized that the contour of PED between the lifted edge of the RPE and Bruch membrane could be a predictor for subsequent development of CSC in VKH cases with corticosteroid treatment.

## Methods

Institutional review board approval (No.2020KY (L)-37) was obtained from Tianjin Medical University Eye Hospital (TMUEH) for the retrospective review of clinical records for all VKH patients at the Uveitis & Ocular Immune Department from August 1, 2011, to August 31, 2020. Informed consent was exempt as it was a retrospective study. This research adhered to the tenets of the Declaration of Helsinki. According to the 2001 diagnostic criteria [9], all VKH patients were identified via medical records and were included if OCT presented well-demarcated, abrupt elevations of the RPE with a homogeneously hyporeflective sub-RPE space and diagnosed with PED by two different ophthalmologists. The retrieved data included demographics, the best-corrected visual acuity (BCVA) at baseline and every follow-up visit, details of the ocular and systemic examination, imaging details of OCT scans, FFA and ICGA, therapeutic regimen, treatment response and complications, long-term anatomical outcomes, and clinical outcomes.

**Fig. 1:**
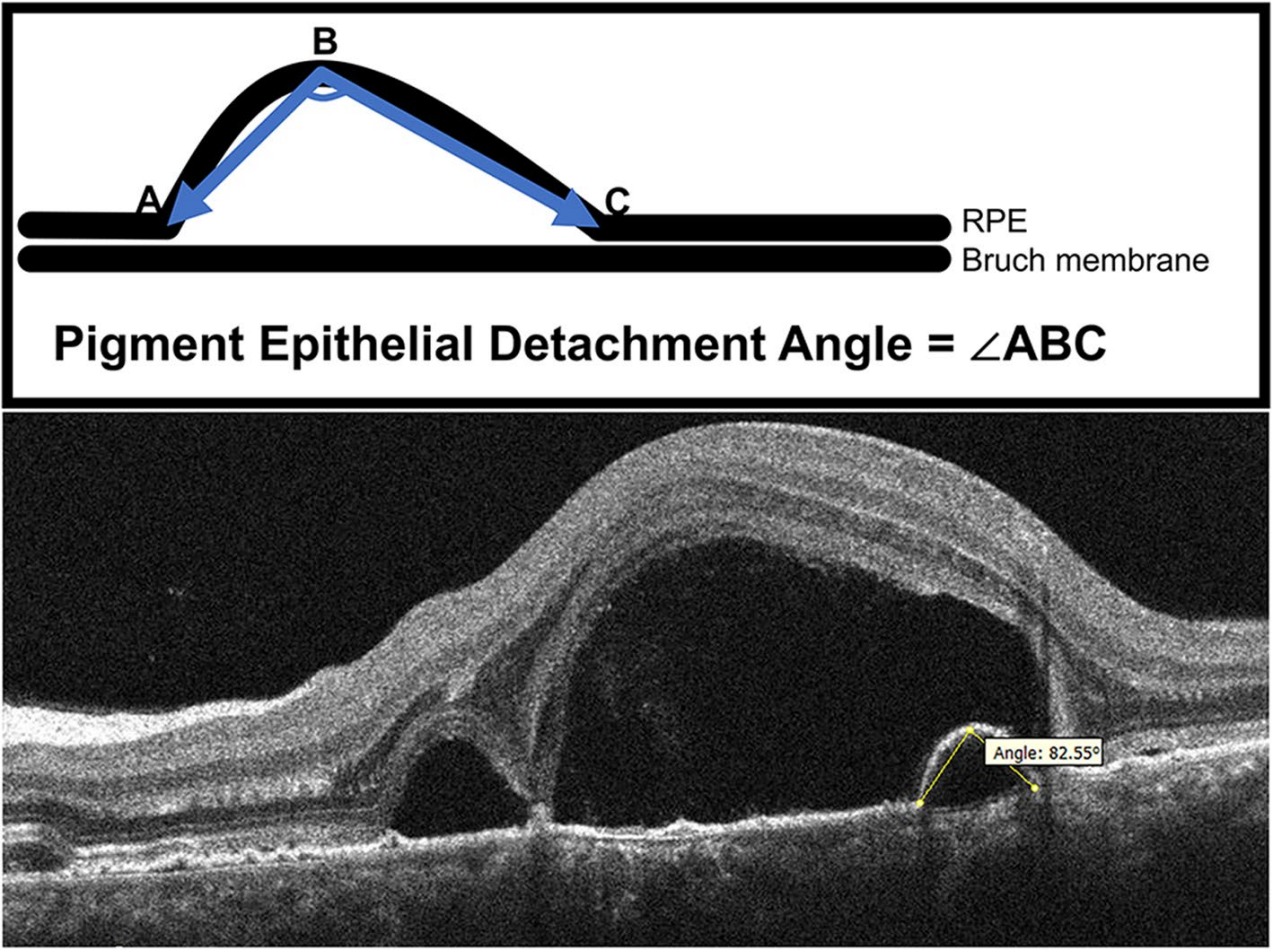
The retinal pigment epithelial detachment angle (PEDA). (Top) Diagram showing the PEDA, the angle between the two lines of the vertex of lifted RPE to the two edge points of the Bruch membrane. (Bottom) Optical coherence tomography (OCT) cross-sectional image of PEDA

We conceived PED angle (PEDA) based on the contour of PED as a useful parameter for predicting the prognosis of PED, under the enlightenment of the macular hole angle [10]. The PEDA was defined as the angle between the two lines of the vertex of the lifted RPE to the two edge points of the Bruch membrane (Fig. 1). PEDA was measured using Digimizer software (Version 5.4.4). For standardization purposes, the average of three measurements was used for analysis. Eyes were classified into two groups: CSC and non-CSC groups. The PEDA and clinical outcomes for each group were evaluated and recorded. The CSC diagnosis criteria in our VKH patients included: there was no active inflammation in both anterior chamber and vitreous cavity when ERD reoccurred; FFA disclosed the intense leakage at the RPE level with an “ink-blot’’ or “smokestack” pattern, which was colocalized with the neurosensory retinal detachment on OCT; slight hyperfluorescence and leakage was allowed on FFA, due to the incomplete resolution of intraocular inflammation in VKH; the VKH patient responded well to the corticosteroid treatment before CSC development, and the neurosensory retinal detachment resulted from CSC could subside with the cessation of corticosteroid therapy.

The SPSS program was used for statistical analysis. BCVA measurements were transferred to the logarithm of the minimum resolution angle (LogMAR) for statistical analysis. Descriptive statistics included mean and standard deviation for normally distributed continuous variables as well as median and range for non-normally distributed continuous variables. Owing to the small sample sizes in our study, all statistical analyses were conducted using the nonparametric Wilcoxon (paired samples) or Mann–Whitney (independent samples) signed rank sum tests. Statistical significance was set at *P* < 0.05.

## Results

A total of 470 eyes (235 patients) with VKH were identified, 44 patients were diagnosed with complete VKH disease, 144 with incomplete VKH, and 77 with probable VKH. The population consisted of 128 female (54.5%) and 107 male (45.5%) patients aged a mean of 46.7 ± 15.6 years (range: 7–85). The most frequent extraocular finding was meningismus. The characteristic fundus manifestations were multiple ERDs at the posterior pole and optic edema. Auxiliary examinations were used to provide aid for diagnosis and evaluate relevant changes in eyes. FFA revealed multifocal regions of pinpoint leakage, pooling, and staining of optic disc. ICGA disclosed numerous hyperfluorescent areas and hypofluorescent dark dots. OCT presented serous retinal detachment with choroidal thickening, and the typical manifestations of RPE were undulating RPE line and RPE bumps. Based on empirical findings, first choice of treatment was systemic high dose corticosteroids. For those patients who were intolerant or resistant to corticosteroids, immunosuppressive agents and/or biological agents were employed to suppress the ocular inflammation.

A total of 12 eyes (10 patients) developed serous PED (12/470, 2.55%), it occurred in 10 eyes (8 patients) at the onset of VKH, and in other 2 eyes (2 patients) during the treatment follow-up. The demographic and clinical data of the 12 eyes (10 patients) are summarized in Tables 1 and 2. In the 10 eyes with PED occurring at the onset of the disease, it gradually resolved in 6 eyes, and developed into CSC in the remaining 4 eyes. Among the 4 eyes with CSC development, the CSC occurred after the resolution of inflammation in both anterior chamber and vitreous cavity in two eyes (case 5 and case 9), on the other hand, the CSC were considered to occur simultaneously with initial PED and VKH in another two eyes (bilateral eyes of case 10). Over the course of systemic corticosteroid therapy, there were two VKH eyes developed PED, but it gradually resolved in one eye and developed into CSC in the other one. Overall, fundus photography, OCT, and FFA images indicated that 5 eyes of 4 VKH patients with PED experienced superimposed CSC (5/470, 1.06%). Interestingly, OCT revealed that the all the neurosensory retinal detachments of CSC colocalized with PEDs; meanwhile, the microrips of RPE at the margin of PED was clearly seen in the bilateral eyes of case 10, in which the CSC, PED and VKH were considered to occur simultaneously.

**Table 1:** Demographics of VKH patients associated with PED

Chraterastics	Value
Number of patients / eyes	10 / 12
Number of right / left eye	7 / 5
Number of males / females	6 / 4
Prevalence of PED	2.55% (12/470)
Age (years)	49 ± 11.9
Average follow-up time (months)	10.5 ± 5.2 (3–18)
BCVA at baseline (LogMAR) (median and range)	0.45 (0.30–1.00)
BCVA at last visit (LogMAR) (median and range)	0.08 (0.00–0.45)
*P* value of BCVA improvement	0.02

*PED* Retinal pigment epithelial detachment, *VKH* Vogt-Koyanagi-Harada syndrome, *BCVA* Best corrected visual acuity

Normally distributed continuous variables are shown as mean and standard deviation and non-normally distributed continuous variables as median and range

**Table 2:** Clinical characteristics of VKH patients

Case	Age / Gender	Eye	BCVA at baseline	BCVA at final visit	Follow up (months)	The time of PED occurrence	PEDA (degree)	CSC occurrence	Time between PED and CSC	PED outcomes	Treatment
1	46 / F	OD	20/50	20/20	10	Initial visist	109.80	No	-	Regression	Routine oral steroid tapering
2	49 / F	OD	20/200	20/20	13	Initial visist	101.82	No	-	Regression	Routine oral steroid tapering & AZA
3	34 / F	OD	20/63	20/20	12	Initial visit	112.93	No	-	Regression	Routine oral steroid tapering
		OS	20/63	20/20	12	Initial visit	63.61	No	-	Regression	Routine oral steroid tapering
4	60 / F	OS	20/40	20/33	7	Initial visit	133.87	No	-	Regression	Routine oral steroid tapering
5 (Fig. 2)	40 / M	OS	20/200	20/20	10	Initial visit	83.18	Yes	3 months	Persistence	Rapid oral steroid tapering & MMF subthreshold micropulse laser photocoagulation & continue-wave laser photocoagulation
6	30 / F	OD	20/40	20/40	18	During follow-up	89.60	Yes	6 months	Regression	Rapid oral steroid tapering & CsA & AZA & subthreshold micropulse laser photocoagulation
7	58 / F	OD	20/2000	0.2	6	Initial visit	125.73	No	-	Regression	Intravenous methylprednisolone & oral tapering steroid & CsA
8	62 / M	OS	20/22	20/20	6	During follow-up	100.33	No	-	Regression	Oral tapering steroid
9	64 / M	OD	20/200	20/28	9	Initial visit	59.92	Yes	2 months	Regression	Rapid oral steroid tapering & MMF
10 (Fig. 3)	47 / F	OD	20/33	20/63	8	Initial visit	78.89	Yes	0	Regression	Rapid oral steroid tapering off & CsA & MMF & ADA & subthreshold micropulse laser photocoagulation
		OS	20/40	20/63	8	Initial visit	76.88	Yes	0	Regression	Rapid oral steroid tapering off & CsA & MMF & ADA & subthreshold micropulse laser photocoagulation

*F* Female, *M* Male, *PED* Retinal pigment epithelial detachment, *PEDA* PED angle; CSC, central serous chorioretinopathy, *AZA* Azathioprine, *CsA* Ciclosporin A, *ADA* Adalimumab, *MMF* Mycophenolate mofetil

We divided the 12 eyes into two groups according to whether CSC developed or not: the CSC group and the non-CSC group. The average time taken between PED and CSC occurrence was 2.2 ± 2.5 months (range 0–6 months). Upon CSC diagnosis, corticosteroid was rapidly tapered off, and alternative therapies were applied, including immunosuppressants and biologics (AZA, CsA, MMF, ADA), continuous-wave laser photocoagulation for outside foveal leakage, and subthreshold micropulse laser photocoagulation (577 nm) for foveal leakage. After the treatment was adjusted, all PED and subretinal fluid resolved completely, except for the PED in case 5.

BCVA with a statistical difference was observed between the initial visit and the last follow-up in the 12 eyes (*P* = 0.02) (Table 1). There was no difference in BCVA at baseline between the two groups, but BCVA improvement until the last visit in the non-CSC group was greater than that in the CSC group, although the difference was not statistically significant due to the small sample size (*P* = 0.25) (Table 3). The PEDA values ranged from 59.92 to 133.87 in the 12 eyes, which in the CSC group was significantly smaller than that in the non-CSC group (77.69 ± 11.06 VS 106.87 ± 22.62, *P* = 0.03) (Table 3).

**Table 3:** Parameters of CSC group and non-CSC group

Parameters	CSC group	Non-CSC group	*P* value
PEDA	77.69 ± 11.06	106.87 ± 22.62	0.03
BCVA at baseline	0.30 (0.25–1.00)	0.50 (0.30–1.00)	0.68
BCVA improvement	-0.27 ± 0.61	-0.55 ± 0.46	0.25

*PEDA* Retinal pigment epithelial detachment angle, *BCVA* Best corrected visual acuity, *CSC* Central serous chorioretinopathy

Representative three eyes of CSC group with clear choroidal morphology on OCT recordings were displayed here.

## Case 5

**Fig. 2:**
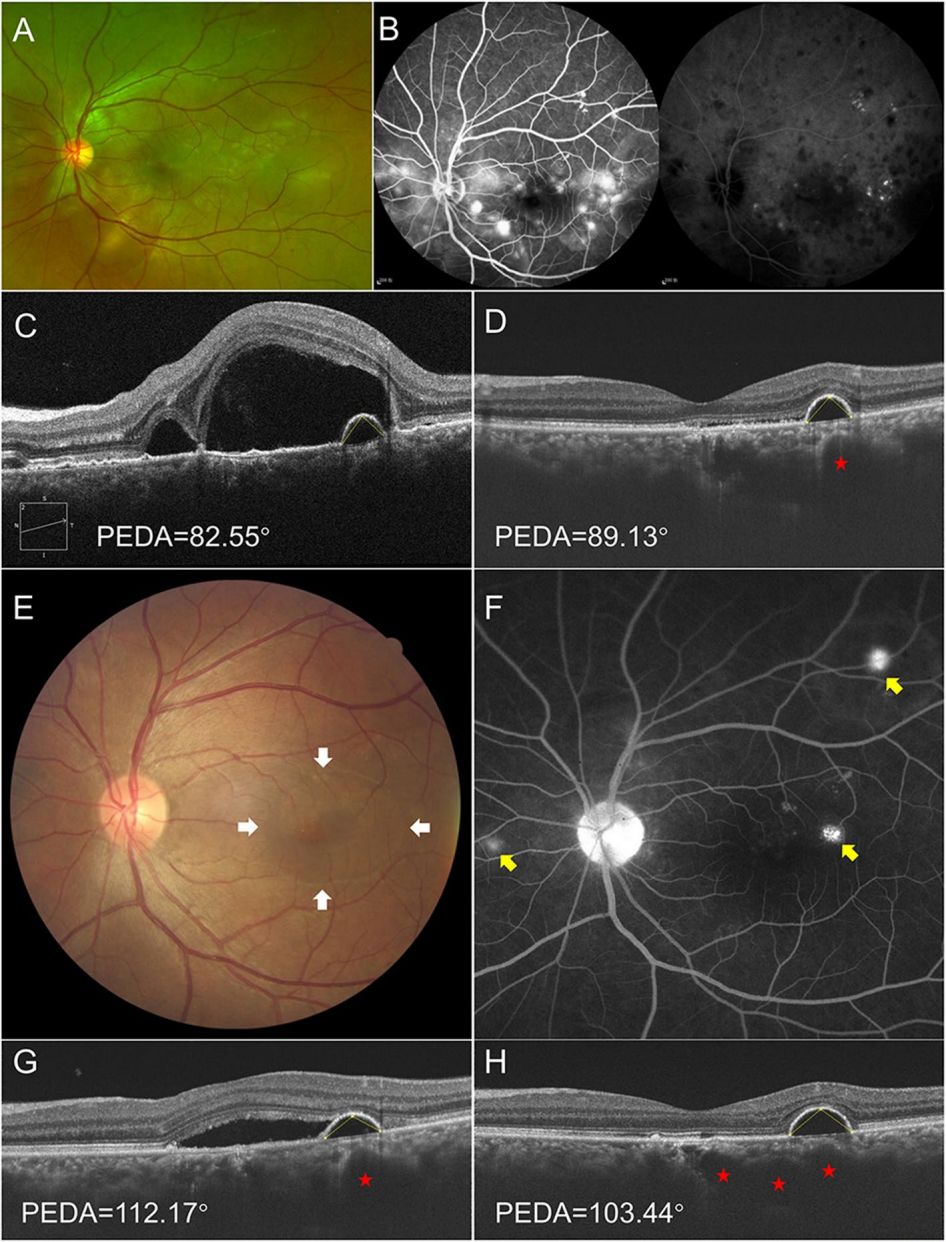
Disease progression and outcomes of case 5. **A-C** Multimodal imaging examinations of case 5 at initial indicating the diagnosis of Vogt-Koyanagi-Harada disease (VKH). **A** Wide field fundus photography demonstrated multiple exudative retinal detachment (ERD) in the left eye. **B** Fundus fluorescein angiography (FFA) and indocyanine green angiography (ICGA) revealed typical acute VKH. **C** OCT presented multilobular ERD and hyperreflective dots, a single PED, and undulating RPE. The PED angle (PEDA) was 82.55°.**D **OCT, taken 3 weeks after systemic corticosteroid treatment, showed a quiet shallow neurosensory retinal detachment, the thinned inner choroidal layer and dilated large choroidal vessel (red pentagram) beneath the persisted PED in the left eye. The PEDA increased to 89.13°. **E–G** Development of central serous chorioretinopathy (CSC) during the systemic corticosteroid treatment period. **E** Fundus photography showed dome-shaped macular (white arrows). **F** Multiple leakages with “ink-blot’’ pattern was noted on FFA (yellow arrows). **G** OCT showed sensory retinal detachment corresponding dome-shaped macular, persisted PED, thinned inner choroidal layer and dilated large choroidal vessel (red pentagram). The PEDA increased to 112.17°. **H** Persisted PED and dilated choroidal large vessels (red pentagrams) were noted on OCT after macular subretinal fluid of CSC resolved completely at the last follow-up. The PEDA of the persisted PED was 104.44

A 40-year-old man presented with progressive vision loss in both eyes accompanied by a headache for five days. His BCVA was 20/50 in the right eye and 20/200 in the left eye. Fundus photography revealed multiple bubble-like elevations at the posterior pole in both eyes (Fig. 2A). FFA revealed optic disc hyperfluorescence and multiple pinpoint leakage at the level of RPE, with subsequent pooling of dye in the subretinal space of both eyes. ICGA showed bilateral hypofluorescent areas corresponding to overlying ERD, multiple hypofluorescent dark dots and disseminated spotted choroidal hyperfluorescence, and blurred choroidal vessels (Fig. 2B). OCT presented multilobular ERD and hyperreflective dots, colocalized PED, and undulating RPE in the left eye (Fig. 2C). A diagnosis of acute VKH disease was made based on extraocular and ocular manifestations. The multiple ERD gradually subsided, and ocular inflammation was controlled under systemic prednisone treatment with a starting dose of 1 mg/kg/day followed by gradual tapering. Three weeks after presentation, BCVA improved to 20/20 in the right eye and 20/25 in the left eye. Only a slit-like neurosensory retinal detachment on OCT remained in the left eye, without any changes in PED; meanwhile, the thinned inner choroidal layer and dilated large choroidal vessel under the PED was noticeable (Fig. 2D). However, dome-shaped neurosensory retinal detachment reoccurred in the macular region at the two-month follow-up, while both the anterior chamber and vitreous cavity were quiet (Fig. 2E), FFA showed multiple punctate leakages in the left eye, which increased in size and intensity as the angiogram progressed (Fig. 2F), and barely visible hyperfluorescent spots in the right eye, OCT revealed neurosensory retinal detachment of the macular region and colocalized PED, the thinned inner choroidal layer and dilated large choroidal vessel under the PED (Fig. 2G). A diagnosis of steroid-induced multiple CSC was made. Oral prednisone was rapidly tapered to a low dose, and mycophenolate mofetil was added. Continuous-wave laser photocoagulation was performed for outside fovea leakage, and subthreshold micropulse laser photocoagulation was used for foveal leakage. One month after the adjusted therapy, bilateral BCVA was 20/20, and complete resolution of subretinal fluid was observed on OCT of the left eye; however, the PED, thinned inner choroidal layer and dilated large choroidal vessel persisted until the end of follow-up (Fig. 2H).

## Case 10

**Fig. 3:**
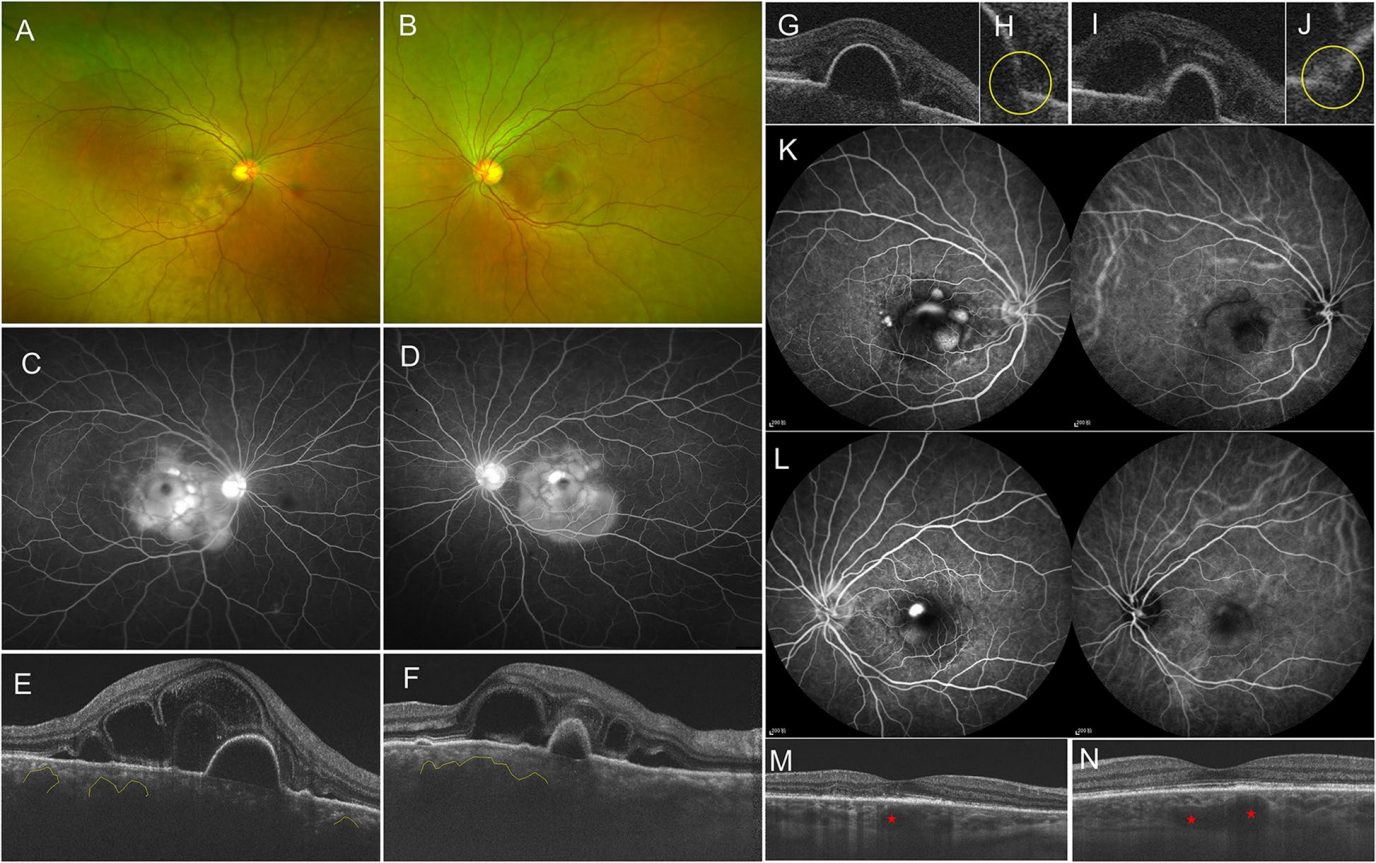
Disease progression and outcomes of case 10. **A-J** Multimodal imaging examinations of case 5 at initial indicating the diagnosis of VKH and CSC. Widefield fundus photos (**A **and** B**) of acute VKH. Widefield late-phase fluorescein angiography of acute VKH (**C **and** D**). **E-J** OCT of revealed the presence of subretinal fluid, septae, PED, dilatated large choroidal vessels (yellow polyline) and RPE microrips (yellow circle). **K-L** Three weeks after treatment with rapid and aggressive high-dose systemic corticosteroid, adalimumab and mycophenolate mofetil, FFA demonstrated ‘ink-blot’ and ICGA showed large choroidal venous dilatation. **M–N** Eight months after the presentation, OCT showed complete remission of neurosensory retinal detachment and PED, and dilated large choroidal vessels in bilateral eyes (red pentagrams)

A 47-year-old woman presented with bilateral acute vision loss for 1 day, accompanied by metamorphopsia and headache. She delayed prior systemic or ocular history. On examination, BCVA was 20/32 in the right eye and 20/40 in the left eye. The anterior segment examination showed bilateral shallow anterior chamber. Ultrasound biomicroscopy revealed bilateral ciliary body detachment. Fundus examination revealed multiple ERDs at the posterior pole in both eyes (Fig. 3A-B). The wide-field FFA demonstrated bilateral optic disc hyperfluorescence and multilobular dye pooling in the late period (Fig. 3C-D). On OCT, serous subretinal fluid accumulation with subretinal septa, hyperreflective dots, and multiple PEDs were evident in both eyes. Simultaneously, the attenuation of inner choroidal vessels below the RPE and dilation of the outer large choroidal vessels below PED were prominent (Fig. 3E-F). Based on the above clinical examinations, a provisional diagnosis of acute VKH was made and the patient was treated with 500 mg/day of methylprednisolone intravenously for 3 days, followed by oral prednisolone. The number of posterior multiple ERDs decreased significantly and optic disc swelling improved in 3 weeks. However, there was no improvement in some certain ERDs and PEDs, and the patient’s BCVA continuously deteriorated to hand movement in the right eye and 20/160 in the left eye, despite the addition of ADA and MMF. An FFA combined with an ICGA was performed. Compared with her first FFA, the classic features of VKH that late optic disc staining and multilobular dye pooling disappeared, but the multifocal leakage at the macula followed by pooling into the subretinal space in the form of an ‘ink-blot’ was present, and the location of the ‘ink-blot’ coincided with the intense hyper-fluorescence on her first FFA. ICGA showed apparent dilatation of large choroidal vessels (Fig. 3K-L). Furthermore, we carefully checked the OCT performed with the line scan pattern of 256 images at her first visit and found obviously microrips of the RPE at the margin of the PED (Fig. 3G-J). These findings strongly suggest that VKH and CSC might have simultaneously occurred in this patient. Subsequently, oral prednisone taper was accelerated and withdrawn in 15 days, adalimumab and mycophenolate mofetil were maintained, cyclosporine was added, and the subthreshold micropulse laser was used to seal the RPE microrips. The persisted ERDs and PEDs in both eyes subsided gradually in one month. On the last examination, eight months after the disease onset, BCVA improved to 20/63 in both eyes. OCT indicated complete remission of neurosensory retinal detachment and PED in both eyes, but the thinned inner choroidal layer and dilated large choroidal vessel still existed in bilateral eyes (Fig. 3M-N).

## Discussion

To the best of our knowledge, the association between PED, CSC, and VKH has not been reported. For the first time, our study revealed that the occurrence of PED might be associated with CSC development in VKH cases. PED with high choroidal hydrostatic pressure predisposes to the later development of CSC in VKH cases with corticosteroid therapy. Notably, our results indicated that PEDA might be a significant predictive factor for CSC development in VKH cases.

CSC can develop following the administration of corticosteroids via diverse routes [11]. Corticosteroids could elicit or aggravate CSC by inducing choroidal enlargement, which may be caused by the inappropriate mineralcorticoid receptor activation and upregulation of endothelial vasodilatory potassium channel KCa2.3 (calcium-dependent channel) [12]. However, the pilot study demonstrated that steroid-induced CSC might be an idiosyncratic response in selected vulnerable individuals rather than a dose-dependent effect [13]. CSC induced by corticosteroids has been reported to occur in a wide variety of ocular inflammatory conditions but with a relatively low incidence. Majumder et al. revealed that the prevalence of CSC in uveitis was approximately 0.13% in a large retrospective study involving 22,721 patients in India [14]. We found that the specific incidence rate of CSC in VKH was 1.06% in our cohort of 235 patients. This rate is much higher than that revealed in the Indian uveitis population, which included patients with all types of uveitis.

VKH disease and CSC are considered to be completely different diseases with different pathophysiologies. However, CSC may clinically mimic VKH or develop in VKH cases. Hence, it is of paramount importance to differentiate CSC from the relapse of inflammation once dome-shaped neurosensory retinal detachment reoccurs or worsens in VKH, as the management of these two conditions is paradoxical. Delayed diagnosis of CSC lead to permanent retinal damage with subsequent poor visual prognosis [15, 16]. Multimodal imaging examinations are useful in discriminating CSCs from VKH. On OCT, fluctuations of ILM, subretinal septa, subretinal membranous structures and hyperreflective dots, bulges of RPE, folds of RPE, and a resultant high RPE undulation index are characteristics of VKH. However, isolated serous PED always points to CSC because of its high prevalence in CSC, and it is regarded as an intermediate stage between pachychoroid and classic CSC [15, 17–20]. Meanwhile, mechanical disruption at the RPE level is the most plausible factor responsible for generating the focal leakage points in CSC [21]. The subfoveal choroidal thickness increases markedly during the acute phase of both VKH and CSC, and it is thicker in acute VKH than in acute CSC [17, 19]. The thick choroid of acute VKH is diffuse and homogenous and the infiltration of inflammatory cells and the proteinaceous fluid exudates may obscure the choroidal vessel margin [22]. Nevertheless, the choroidal thickening of CSC is considered to result from a thinned inner choroidal layer and enlarged underlying hyporeflective choroidal lumina [22–24]. The typical FFA manifestations of VKH are numerous punctate hyperfluorescent dots at the RPE level in the early stage, followed by staining and pooling of the dye in the subretinal space, optic disc hyperfluorescence, and leakage in the late stage [3]. Nonetheless, in classic CSC, unifocal leakage at the RPE level with an “ink-blot” or “smokestack” pattern is typical, but in eyes with steroid-induced CSC, the intense leakage from multiple regions is often seen [15, 25]. Typical ICGA signs of VKH include early hyperfluorescent stromal vessels, late hypofluorescent dark dots, and fuzzy or lost vascular patterns of large stromal vessels due to choroidal inflammation [1–3, 26]. Whereas geographic areas of hyperfluorescence with blurred contours are the hallmark of choroidal hyperpermeability in CSC [15].

The explicit pathogenesis of VKH and CSC remains poorly understood. In acute VKH, the diffuse and homogenous choroidal inflammation leads to multiple blood-retinal outer barrier dysfunctions, so multiple accumulations of fluid with the abundant protein under the neurosensory retina occur, which are distinctive signs of acute VKH [1, 27].. Occasionally, the unusual sign of PED occurs in acute VKH cases, whose pathophysiology is speculated to be composed of hyperpermeability of choroidal vessels resulting from choroidal inflammation and the relatively high hydrostatic pressure across the swelling choroidal vascular bed [7]. In contrast, high hydrostatic pressure in the choroid is believed to be the primary underlying pathophysiology in classic CSC, which pushes the RPE forward to relieve pressure and causes RPE detachment from the Bruch membrane [28]. The PED enlarges as the pressure further increases, which will add angulation and mechanical stress to the base of the PED and may weaken or rupture the RPE monolayer layer [28, 29]. The prospective study of acute CSC indirectly confirms these pathological processes, and transverse and RPE fit C-scans matching the leakage site were superimposed on an OCT fundus photograph and linked to the leakage location on the FFA, in which PED was observed in 85% of eyes and RPE microrips were detected in 54.5% of eyes [21]. Once the excessive hydrostatic pressure is released, there are no more persistent triggers in CSC. Therefore, the possibility of spontaneous resolution of the serous neurosensory retinal detachment is relatively high, and observation is the main treatment modality. As Gupta et al. reported in the natural course of CSC, RPE microrips close spontaneously [21].

Interestingly, VKH and CSC seemed to occur simultaneously in case 10 before corticosteroid application since multimodal images revealed characteristic changes in both VKH and CSC at her first visit, just one day after the disease onset. On the second FFA, both multiple subretinal staining and optic disc hyperfluorescence disappeared after corticosteroid therapy, indicating that the high-dose systemic corticosteroid has effectively suppressed acute intraocular inflammation. Meanwhile, the distinctive signs of CSC are also obvious, and the patient’s vision even continuously deteriorates. The leakage points with ‘ink-blot’ on the second FFA coincide with the intense hyper-fluorescence of the first FFA, and ICGA shows the apparent dilatation of large choroidal veins. The persisted ERDs and PED in both eyes subsided gradually after one month of discontinuation of oral prednisone, continuous treatment of immunosuppressants and biologics, and the subthreshold micropulse laser for RPE microrips. Importantly, there was no recurrence of ocular inflammation or neurosensory retinal detachment throughout the whole follow-up. These results suggest that VKH and CSC occurred simultaneously in case 10 at the onset of the diseases. According to this case, we can speculate that in other VKH and PED patients, the initial PED may result from a combination of two distinct mechanisms: inflammation of VKH and high hydrostatic pressure. The PED resolves with high-dose systemic corticosteroid treatment if the main cause is inflammation of VKH, or develops into classic CSC once high hydrostatic pressure is the main cause.

PED is a common sign in CSC, in which the prevalence of PED with or without associated ERD is reported to be 44.2–100% [15, 17–19]. Conversely, PED is observed in 0–10% of eyes with acute VKH disease, according to several cross-sectional OCT-based studies [17–20, 30]. The prevalence of PED in acute VKH patients in our study is 2.55% (12/470). PEDs resolved with corticosteroid therapy in 7 eyes, while in the other 5 eyes PEDs persisted and colocalized classic CSC developed during the treatment course. To further determine the relationship between PED and CSC development in VKH patients, we proposed the PEDA, an index reflecting the PED deformation and hydrostatic pressure, to predict the outcomes of PED. The calculation of PEDA is simple and can be easily applied clinically. Since the PEDA formed between the lifted RPE and Bruch membrane represents primarily superoinferior stretches, a lower PEDA value indicates a smaller horizontal and greater perpendicular PED dimension, implying a higher hydrostatic pressure and more RPE stretches. Therefore, once the hydrostatic pressure further increases under the treatment of systemic corticosteroid, patients with small PEDA may be more likely to develop defects of the RPE monolayer to relieve pressure, which is the classic CSC. Our measurement values of initial PEDA demonstrate the hypothesis, in which the PEDA values in the CSC group were significantly smaller than that in the non-CSC group. Moreover, when comparing the PED before and after CSC development in Case 5, we can obviously identify that the pressure within the PED was released after the onset of CSC, the angle of the PED became obtuse and the value of PEDA increased. For VKH patients with small PEDA, immunomodulatory and/or biologicals could be the first choice. If the therapy of rapid and aggressive high-dose systemic corticosteroid along with immunomodulatory and/or biologicals is adopted, close follow-up will be necessary to monitor the changes in PED and the occurrence of classic CSC, and timely adjustment of the therapeutic regimen may lower the risk of vision impairment. In our cohort, the BCVA improvement of the CSC group tended to be less than that of the non-CSC group, although there was no statistical difference due to the small sample size, indicating that the early diagnosis of CSC is important for saving vision.

Discontinuation of corticosteroids is suggested as the first step in the treatment of CSC in ocular inflammatory conditions, and adding immunosuppressants and/or biological agents may be required to control ocular inflammation [14, 31]. CSC leakage points outside the fovea could be treated with continuous-wave laser photocoagulation [14]. In our series, four CSC eyes received subthreshold micropulse laser photocoagulation for foveal leakage and yielded favorable results. In contrast to the traditional continuous-wave laser, the subthreshold micropulse can provide the required activation energy of the RPE cells without damaging the retina [32, 33].

The retrospective nature of the present study brings some limitations. First, these results only provided preliminary conclusions due to a small sample size and low incidence. Second, the application of PEDA was restricted since it could not be calculated in cases where severe vitreous opacities, mature cataracts, or other conditions that prevent the presence of clear OCT recordings. Third, details of the choroid cannot be obtained in every case due to the limited penetration of OCT and poor delineation of the deeper posterior border of the choroid, and it is better to use enhanced depth imaging mode scans of OCT for precise measurement in future clinical cases. Despite its limitations, the study certainly adds to our understanding of the associations between PED in VKH patients and CSC development. Further research is needed to confirm the predictive value of PEDA and to clarify the mechanism of classic CSC development in VKH cases.

## Conclusion

The present study provides the first assessment of PED and its relationship with CSC development in VKH patients. Our results indicate that PED predisposes to the development of CSC in selected VKH eyes, systemic corticosteroid treatment may promote the occurrence of classic CSC. We believe that PEDA is a simple parameter to present PED configuration and hydrostatic pressure, it might be an efficient predictive factor for the subsequent development of classic CSC.

## Data Availability

The data and materials are available upon request from the corresponding author at xzhang08@tmu.edu.cn.

## Abbreviations

VKH: Vogt-Koyanagi-Harada
ERD: Exudative retinal detachments
FFA: Fundus fluorescein angiography
ICGA: Indocyanine green angiography
OCT: Optical coherence tomography
RPE: Retinal pigment epithelium
PED: Pigment epithelial detachment
CSC: Central serous chorioretinopathy
TMUEH: Tianjin Medical University Eye Hospital
BCVA: Best-corrected visual acuity
PEDA: PED angle
AZA: Azathioprine
CsA: Cyclosporin A
MMF: Mycophenolate mofetil
ADA: Adalimumab
